# Global Co-Existence of Two Evolutionary Lineages of Parvovirus B19 1a, Different in Genome-Wide Synonymous Positions

**DOI:** 10.1371/journal.pone.0043206

**Published:** 2012-08-13

**Authors:** Marijke W. A. Molenaar-de Backer, Vladimir V. Lukashov, Rob S. van Binnendijk, Hein J. Boot, Hans L. Zaaijer

**Affiliations:** 1 Department of Blood-Borne Infections, Sanquin Blood Supply Foundation, Amsterdam, The Netherlands; 2 Department of Viral Diagnostic Services, Sanquin Blood Supply Foundation, Amsterdam, The Netherlands; 3 Laboratory of Experimental Virology, Department of Medical Microbiology, Center for Infection and Immunity Amsterdam (CINIMA), Amsterdam, The Netherlands; 4 Laboratory for Infectious Diseases and Perinatal Screening, National Institute for Public Health and the Environment (RIVM), Bilthoven, The Netherlands; 5 Laboratory of Clinical Virology, Department of Medical Microbiology, Center for Infection and Immunity Amsterdam (CINIMA), Amsterdam, The Netherlands; University of Texas Medical Branch, United States of America

## Abstract

Parvovirus B19 (B19V) can cause infection in humans. To date, three genotypes of B19V, with subtypes, are known, of which genotype 1a is the most prevalent genotype in the Western world. We sequenced the genome of B19V strains of 65 asymptomatic, recently infected Dutch blood donors, to investigate the spatio-temporal distribution of B19V strains, in the years 2003–2009. The sequences were compared to B19V sequences from Dutch patients with fifth disease, and to global B19V sequences as available from GenBank. All Dutch B19V strains belonged to genotype 1a. Phylogenetic analysis of the strains from Dutch blood donors showed that two groups of genotype 1a co-exist. A clear-cut division into the two groups was also found among the B19V strains from Dutch patients, and among the B19V sequences in GenBank. The two groups of genotype 1a co-exist around the world and do not appear to differ in their ability to cause disease. Strikingly, the two groups of B19V predominantly differ in synonymous mutations, distributed throughout the entire genome of B19V. We propose to call the two groups of B19V genotype 1a respectively subtype 1a1 and 1a2.

## Introduction

Parvovirus B19 (B19V) infection can cause disease in humans, such as aplastic crisis, erythema infectiosum (also called fifth disease), arthritis and hydrops foetalis [1]. In adults, B19V infection often is asymptomatic [2]. Some clinical syndromes are associated with the tropism of B19V for erythroid precursor cells [3]–[5]. Infection of these cells induces cell cycle arrest and cell death, which results in a transient, usually sub-clinical decrease in red blood cells [6]. Other symptoms of B19V infection, such as erythema infectiosum and arthritis, are related to the B19V specific antibody response [7], [8]. B19V is a non-enveloped virus with a single stranded 5.6 kb DNA genome. The internal coding sequence (4.8 kb) of the genome is flanked by terminal repeat sequences at both ends. The coding sequence contains two large open reading frames (ORFs), one encoding the non-structural protein NS1, the other encoding structural proteins VP1 and VP2 [9]. In addition, there are three small ORFs, encoding a 11 kDa protein, a 7.5 kDa protein and the putative X protein. Currently there are three genotypes with subtypes known of B19V, respectively genotype 1a, 1b, 2, 3a and 3b [10]. The genotypes show ∼10% nucleotide divergence on the whole genome [11]. The most prevalent B19V genotype in Northern Europe is genotype 1a. Genotype 2 appears restricted to people born before 1970, and genotype 3 predominantly occurs in people from West Africa [1]. Some studies show that B19V genetic diversity may depend on geographical location and the year of isolation in patients [1], [12]. The genetic divergence of B19V can occur by gradual or sudden replacement [12].

In the Netherlands there is a 4-year epidemic cycle for symptomatic B19V infections [13]. In addition, a seasonal variation is present, with most infections occurring between December and July, with a peak in April [14]. In this study we investigated whether B19V sequences of Dutch blood donors show molecular differences which correlate to geographical and temporal differences in the years 2003 to 2009; and we compared these sequences with B19V sequences available in GenBank, and with B19V sequences obtained from Dutch patients, predominantly children, suffering from fifth disease.

## Materials and Methods

### Ethics Statement

The donor and patient sera in the study were de-identified and renumbered using anonymous numbers before the start of the study. The donor samples were collected as part of routine blood donor screening, and the donors consented to parvovirus analysis of the blood samples. The patient samples were collected as part of governmental, national monitoring of exanthematous diseases.

**Table 1:** Primers used for PCR and sequencing of B19V in Dutch blood donors.

Primer	Sequence 5′ to 3′	Location in genome^1^	Reference
NSoFw	ATG GAG CTA TTT AGA GGG GTG	436–456	[17]
B19-854-F	GAA TGT AAC AAA TTT G	649–664	This study
B19-1025-gt1F	AAA TAC TTT AGA GAT GGA G	820–838	This study
B19-GR	ATG CTA GCC TTA GTT CCC TT	1076–1057	This study
P2F	AAA CTA GCA ATT TAT AAA GC	1213–1232	[15]
B19-1596-R	CCA CAT TTT TTA TCA ATC C	1391–1373	This study
P4-F	TTG GTG GTC TGG GAT GAA GG	1537–1556	[15]
B19-1955-R	CCA CGC ATT TTT TGA TCT ACC C	1637–1616	This study
EVF	AAT GCA GAT GCC CTC CAC	1903–1920	[33]
EVR	ATG ATT CTC CTG AAC TGG TCC	2095–2075	[33]
PV2-F	GCT TGG TAT AAT GGA TGG AA	2302–2321	[15]
PV-3R	CCA GAC AGG TAA GCA CAT TT	2423–2404	[15]
PV4-F	TTT GAC TTA GTT GCT CG	2621–2637	[15]
B19-SR	CCA GGC TTG TGT AAG TCT TC	2799–2780	[15]
B19-JR	CAG CTG CAC CTT TTA AAG TA	3053–3034	This study
B19-KF	TTA TAA GGT GTT TTC TCC CG	3283–3302	This study
B19-MF	TTT CAG CTT TTA GGT ACA GG	3379–3798	This study
B19-3717-F	GAT GTT ACA GAC AAA ACW GGA GG	3512–3534	This study
B19-LR	AGC ATA TTG AGG GGG AAA GTA	3679–3659	This study
B19-3966gt1-2R	CAT AAA ATG CTG ATT CTT CAC	3761–3741	This study
B19-4365-R	CAT ATT TAT CWG TGT CCC	4160–4143	This study
B19-OF	TTT CCC AAT AAA GGA ACC CA	4301–4320	This study
B19-PR	TAC TGT CAT AAT TCC CAC	4588–4571	This study
Vp2oRev	TGG GTG CAC ACG GCT TTT GG	4783–4764	[17]

1 Numbering according to B19V GenBank sequence M13178. [9]

**Figure 1:**
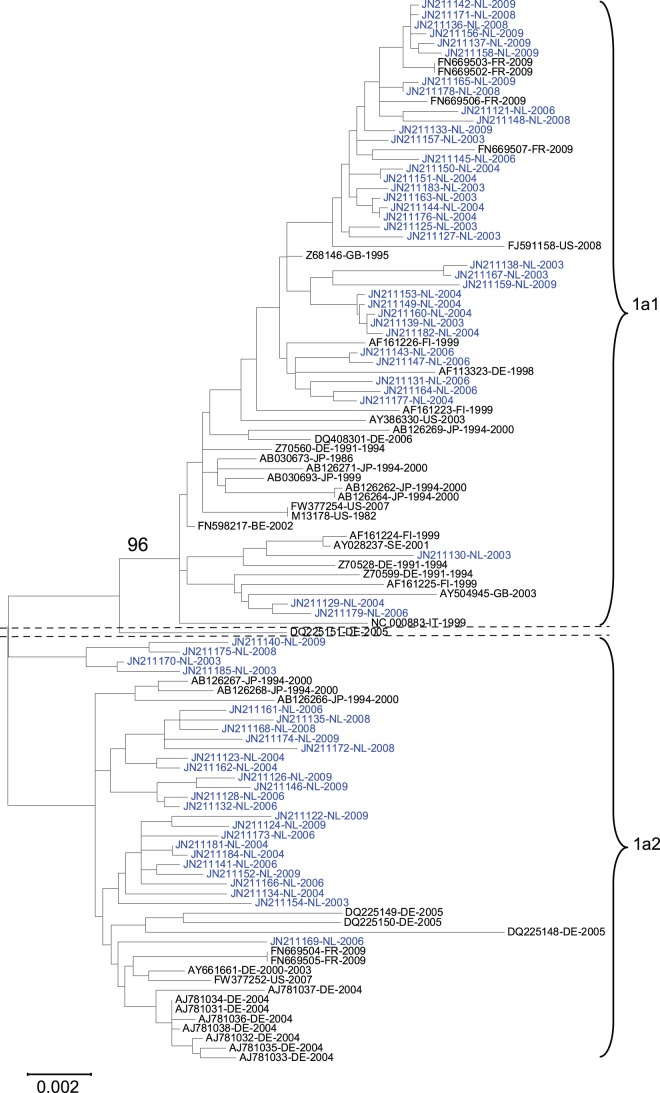
Phylogenetic analysis of Dutch and global B19V 1a strains by Maximum Likelihood method. The tree with the highest log likelihood (−13872,9929) is shown. The bootstrap value for the B19V 1a1 cluster is shown. B19V sequences from blood donors are shown in blue, while global GenBank sequences are shown in black.

### Samples: B19V Infected, Asymptomatic Blood Donors

Blood donations in the Netherlands are routinely screened for presence of B19V DNA, as part of in-process testing for plasma fractionation. Between January 2003 and January 2010 6.5 million blood donations were tested with the Roche LightCycler parvovirus B19 quantification assay. As the Roche test fails to detect B19V genotypes 2 and 3, all donations were also tested with a generic B19V test from 2005 onwards [15], [16]. All blood donations with a B19V DNA load ≥10^6^ IU/mL, reflecting acute parvovirus infection, were collected and stored at −30°C. The epidemiology of B19V infection in this donor population has been described [14]. For this study, a total of 65 B19V positive donor samples were analyzed; namely 14 donations from each of the years 2003, 2006 and 2009; 15 donations from 2004 and 8 donations from 2008. 2006 and 2009 were B19V peak years in the Netherlands. The average load of B19V DNA in the 65 donations was 7.98×10^11^ IU/mL. The median age of the 65 selected donors was 38.7 year (range 18–68).

**Table 2:** GenBank sequences used in this study.

Accessionnumber	Country oforigin	Year ofisolation
AB030673	JP	1986
AB030693	JP	1999
AB126262	JP	1994–2000
AB126264	JP	1994–2000
AB126266	JP	1994–2000
AB126267	JP	1994–2000
AB126268	JP	1994–2000
AB126269	JP	1994–2000
AB126271	JP	1994–2000
AF113323	DE	1998
AF161223	FI	1999
AF161224	FI	1999
AF161225	FI	1999
AF161226	FI	1999
AJ781031	DE	2004
AJ781032	DE	2004
AJ781033	DE	2004
AJ781034	DE	2004
AJ781035	DE	2004
AJ781036	DE	2004
AJ781037	DE	2004
AJ781038	DE	2004
AY028237	SE	2001
AY386330	US	2003
AY504945	GB	2003
AY661661	DE	2000–2003
DQ225148	DE	2005
DQ225149	DE	2005
DQ225150	DE	2005
DQ225151	DE	2005
DQ408301	DE	2006
FJ591158	US	2008
FN598217	BE	2002
FN669502	FR	2009
FN669503	FR	2009
FN669504	FR	2009
FN669505	FR	2009
FN669506	FR	2009
FN669507	FR	2009
FW377252	US	2007
FW377254	US	2007
M13178	US	1982
NC_000883	IT	1999
Z68146	GB	1995
Z70528	DE	1991–1994
Z70560	DE	1991–1994
Z70599	DE	1991–1994

### Near Whole-genome Sequencing of B19V from 65 Blood Donors

From the 65 donor samples B19V DNA was extracted using the NucliSENS EasyMag extractor (BioMerieux) with 1 mL input volume [15]. The elution volume was 40 µL. One µL of the eluate was used for an almost full genome PCR, using the NSoFw and Vp2oRev primers (see Table 1) [17]. The PCR mixture contained 800 µM of each primer, 2 units of long range PCR enzyme, 500 µM of each deoxyribonuleotide triphosphate (dNTP) in 1x Long range buffer containing 2.5 mM MgCl2 (Qiagen). DNA was amplified for 40 cycles; the preheating step, 93°C for 6 min, was followed by 40 cycles of 93°C for 15 s, 58°C for 30 s; 68°C for 6.5 min, and a final elongation step at 68°C for 10 min. After the final elongation the samples were cooled to 4°C. After amplification the PCR products were gel electrophoresed and the concentration was estimated (usually >100 ng/µl). 7.5 µL of the PCR product was used for ExoSAP-IT (USB) reaction according to the manufacturer’s protocol. The purified PCR product was used for Big Dye Terminator cycle sequencing (Applied Biosystems) reactions with B19V specific sequencing primers (see table 1) and 20 ng PCR product/reaction. Sequence analysis was performed on an ABI PRISM 3130xl Genetic Analyser (Applied Biosystems). The B19V sequences from Dutch blood donors were deposited in the GenBank database under accession numbers JN211121 to JN211185.

**Figure 2:**
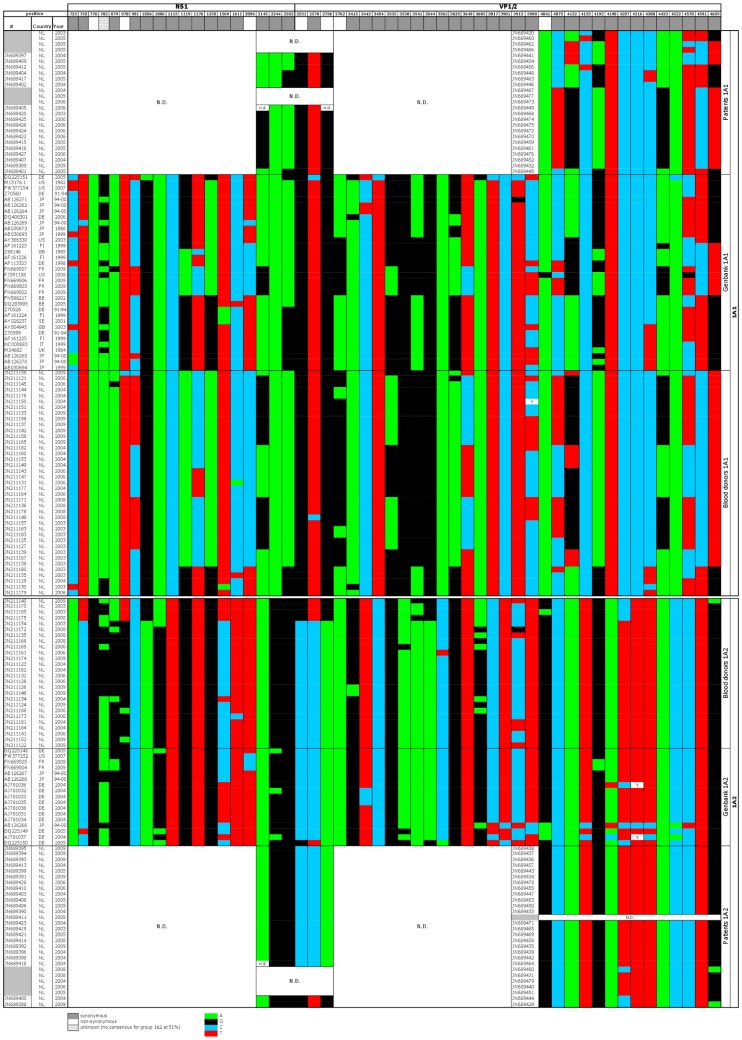
Nucleotide diversity between B19V subtype 1a1 and 1a2. Shown are the variable nucleotides in B19V strains from Dutch blood donors, patients and global GenBank sequences (>4 kb), when a 51% consensus level was used. Along the top of the graph, the position of synonymous mutations (in grey) and the position of nonsynonymous mutations (in white) are depicted, along the B19V genome. In the coloured part each row represents a B19V strain. Strains in the upper part belong to B19V subtype 1a1, strains in the lower part are subtype 1a2. Position 3535–3544 and 4192–4216 are possible substitution hotspots. n.d. = not determined and Y = C or T. green = adenine, black = guanine, blue = cytosine, and red = thymine.

**Figure 3:**
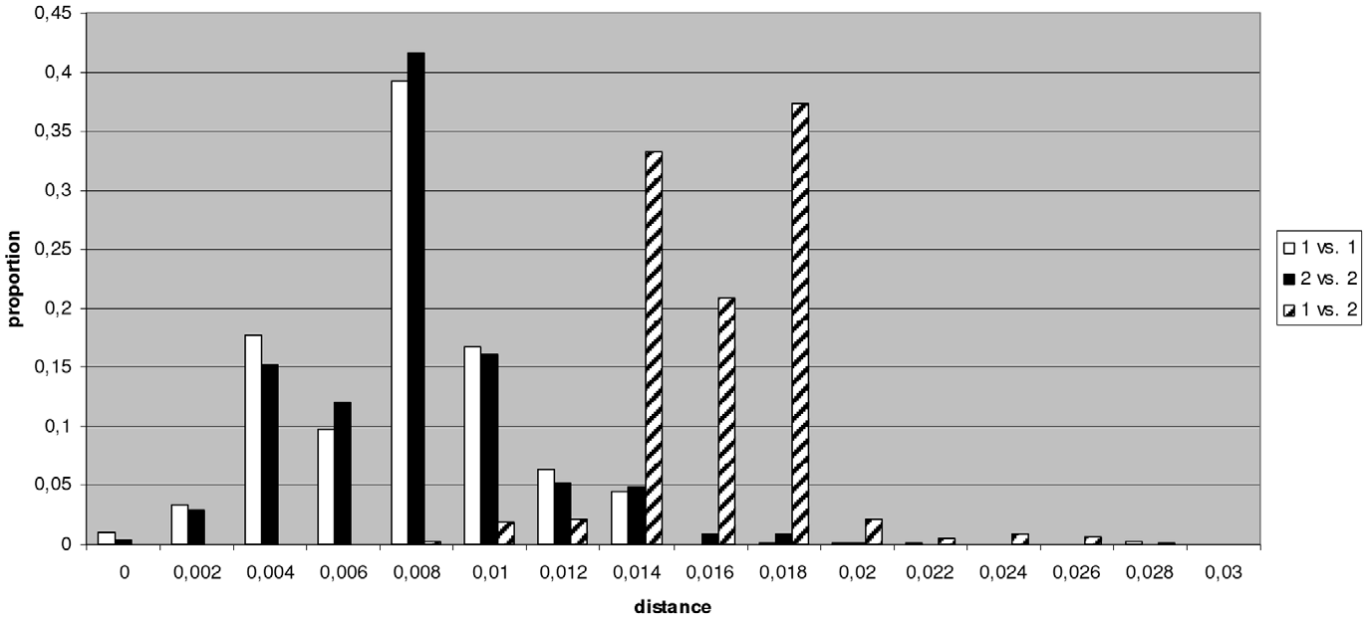
Distribution of intra- and inter-group genetic distances.

### Sequences of B19V from 53 Patients

From 2003 to 2009, samples of patients presenting with rash disease were collected by Dutch municipal health services, and subsequently sent to the National Institute for Public Health and the Environment (RIVM), for laboratory confirmation of B19V infection. The samples were mostly finger-stick blood samples. Fifty-three cases (predominantly children), with signs of fifth disease and testing positive for B19V DNA, were included in this study. The median age of the patients was 6.0 years (range 1–40). Partial B19V DNA sequences were available as follows. For 40/53 patients both a part of the NS1-VP1u region (nucleotide 2112–2763) and a fragment of the VP2 region (nucleotide 3995–4663) were available. The nucleotide position numbering is according to B19V sequence M13178.1. For 1/53 patients only the NS1-VP1u fragment was available; and for 12/53 patients only the VP2 fragment was available. The patient B19V sequences are accessible in the GenBank database via accession numbers JN689388 - JN689428 (for the NS1-VP1u fragment) and JN689429 – JN689480 (for the VP2 fragment).

**Figure 4:**
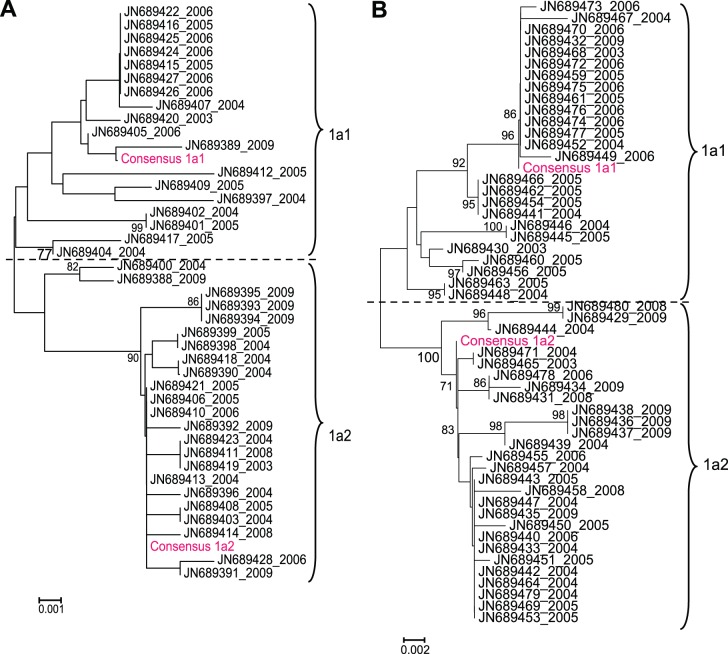
Phylogenetic relationship between the consensus sequences of B19V-1a subtypes found in blood donors (shown in pink) and the sequences found in patients with fifth disease (shown in black). The nucleotide distance relationship is shown for the 652 bp NS1-VP1u region (A) and the 669 bp VP2 (B) region.

### Phylogenetic Analysis and B19V Sequences from GenBank

Sequences were aligned using the ClustalX2 software. Maximum Likelihood analysis was performed using MEGA5 software, using the general time reversible model and gamma-distributed evolution rates among sites (5 discrete gamma categories). Bootstrap analysis was performed (1,000 replicates).

**Figure 5:**
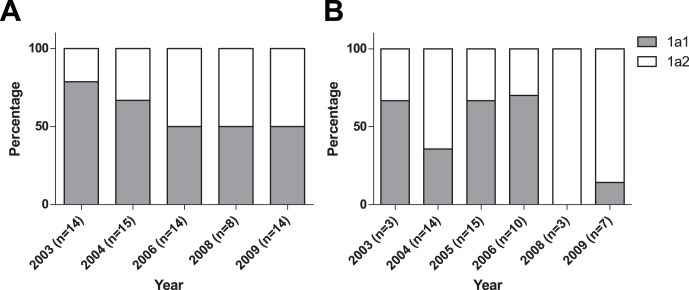
Graph showing the spatial distribution of B19V 1a1 and 1a2 in Dutch blood donors (A) and patients (B) fragment VP2.

In addition, MEGA4 software was used to construct NJ trees, using the neighbor-joining method, pairwise gap deletion, Jukes-Cantor distances for nucleotide sequences and Nei-Gojobori distances for synonymous and nonsynonymous substitutions. Bootstrap analysis was performed with 1,000 replicates. Consensus sequences were generated at different levels, e.g. a 51% consensus level generates a consensus nucleotide when at least 51% of the sequences in a certain group have the same nucleotide at a certain position. If less then 51% of the sequences have the same nucleotide then the consensus nucleotide is unknown (e.g. R = A or G). In the phylogenetic trees the 90% consensus sequences of B19V genotype 1a1 and 1a2 are shown. Nucleotide, synonymous and nonsynonymous distances for pairwise sequence comparisons within and between groups were calculated as for neighbor-joining trees, variance was estimated by bootstrap analysis (1,000 replicates). The dS/dN ratios were calculated in MEGA5. The program used to determine codon usage was previously described [18].

All phylogenetic trees contain the following information: GenBank number- year of isolation; in figure 1 the year of isolation is preceded by the country code.

The genotype 1a B19V GenBank sequences, used to compare the Dutch sequences with global sequences are show in Table 2.

### Geographical Analysis

The geographical distribution of Dutch B19V strains was analysed using MapInfo software 10.5 (Pitney Bowes Business Insight).

## Results

### B19V Screening

Blood donations in the Netherlands are routinely tested for B19V DNA as part of in-process testing for plasma fractionation. Donations containing more than 10^6^ IU/mL B19V DNA are not used for plasma fractionation in order to comply with European regulations, which require that plasma pools used for certain products may not contain more than 10.0 IU/µL B19V DNA. This limit was established to prevent transmission of B19V via plasma products [19]. In the years 2003–2009 6.5 million donations were tested for B19V DNA and 411 donations contained more than 10^6^ IU/mL B19V DNA (0.006% or 1 per 15815 donations) [14]. For this study we have sequenced 65 of these highly viraemic donations, to investigate the molecular epidemiology of B19V in the Netherlands in the period 2003–2009. As expected all 65 donations were positive in the long range PCR and thus could be sequenced.

### Phylogenetic Analysis of B19V in Dutch Blood Donors

All analyzed B19V strains obtained from Dutch blood donors, in the period 2003–2009, belonged to genotype 1a, doubling the amount of B19V sequence information (>4 kb) available for this subtype. The 65 sequences did not contain insertions or deletions when compared to the B19V reference strain PBVBAU (GenBank accession number M13178.1). The B19V nucleotide sequences could be divided into two hitherto unknown groups, see Figure 1. This subdivision was found when considering the complete NS1-VP1/2 fragment (4280 bp, almost the entire coding region; Figure 1), but also when the NS1 region and VP1/2 region were analysed separately (data not shown). Since the ML tree was not different to the NJ tree, due to small actual differences, we used the simpler NJ model in the rest of this study. To investigate if the nucleotide differences between the two groups resulted in differences in protein coding sequence we investigated the synonymous and nonsynonymous mutations. The phylogenetic tree with only synonymous mutations, which were present throughout the entire genome, supported the division in two groups with high bootstrap values (100%, data not shown). On the nonsynonymous level the division was still found, but not statistically supported by bootstrap analysis (data not shown). Thus, there are two groups of B19V genotype 1a simultaneously circulating among Dutch blood donors, these two groups are named B19V 1a1 and 1a2 hereafter.

The B19V donor sequences were used to generate consensus sequences for group 1a1 and for group 1a2, at different consensus levels. The division in two groups was found at every consensus level: 51%, 70%, 80% and 90%. Comparison of the 1a1 51% consensus sequence to the 1a2 51% consensus sequence revealed that 52 nucleotide substitutions, randomly dispersed over the two ORFs, define subtypes 1a1 and 1a2, see Figure 2. The substitutions were mainly synonymous; 44/52 differences were synonymous (85%), 7 were nonsynonymous (14%) and 1 was unknown (AR substitution). The substitutions involved 44/52 transitions (86%) and 7/52 transversions (14%). The number of AG, GA, TC and CT transitions were similar (respectively 13, 9, 9,13) and the number of AT, TA, AC, CA, GT, TG, CG and GC mutations were comparable (respectively 0, 1, 2, 1, 0, 2, 0,1). Thus, there was no evidence for difference in nucleotide preference. There appear to be two substitution hotspots in the VP2 region, at nucleotide positions 3535–3544 and 4192–4216, which did not resulted in different amino acids. The latter hotspot is in an antigenic region [20]. The G to C binding (3 hydrogen bonds) of antibody to the virus in subgroup 1a1 is predominantly changed to the weaker A to T binding (2 hydrogen bonds) in subgroup 1a2, thus perhaps this hotspot reduces the binding of antibodies to B19V. However, the average viral loads between the two subgroups were not significantly different for donors (p = 0.1853) or patients (p = 0.2210).

### B19V 1a1 and 1a2 Sequences Among GenBank Sequences

Subsequently the 1a1 and 1a2 consensus sequences were compared to B19V genotype 1a sequences, as deposited in GenBank (only sequences larger than 4 kb were used). Fifty of the 51 global Genbank B19V 1a sequences fell into the same two groups as present among Dutch blood donors. Again, this difference between the two groups was seen in the analysis of nucleotide sequence (Figure 1) and the synonymous positions, but not in nonsynonymous positions (Figure 2). Only sequence DQ225151 did not cluster with B19V 1a1 or 1a2. Analysis of DQ225151 with Simplot showed that this strain is not a B19V 1a1/1a2 recombinant (data not shown). In conclusion the analysis of B19V sequences reveals a world-wide, simultaneous presence of B19V groups 1a1 and 1a2.

The sequences from blood donors and GenBank were used to calculate genetic differences. The average genetic distances within groups were 0.008 (SE 0.001) for 1a1 and 0.008 (SE 0.000) for 1a2. The mean genetic distance between the two groups was 0.016 (SE 0.001). The distribution of the genetic distances for pairwise sequence comparisons within and between groups is shown in Figure 3.

In addition, the dS and dN were calculated for ORF1 (NS1) and ORF2 (VP1/2). For ORF1 the dS was 0.022 (SE 0.004) for 1a1, 0.024 (SE 0.003) for 1a2 and 0.046 (SE 0.009) for 1a1 vs 1a2. The respective dN values were 0.003 (SE 0.001) for 1a1, 0.002 (SE 0.000) for 1a2 and 0.003 (SE 0.001) for 1a1 vs 1a2. The resulting dS/dN ratios for ORF1 were 7.33 for 1a1, 12.00 for 1a2 and 15.33 for 1a1 vs 1a2.

For ORF2 the dS/dN ratios were higher, namely 34.00 for 1a1 (dS: 0.034 (SE0.004) dN: 0.001 (SE 0.000)), 15.50 for 1a2 (dS: 0.031 (SE 0.003) dN: 0.002 (SE 0.000)) and 23.66 for 1a1 vs 1a2 (dS: 0.071 (SE 0.008) dN 0.003 (SE 0.001)). These high dS/dN ratios for B19V subgroups suggest negative selection.

### B19V in Patients with Erythema Infectiosum

Shorter sequences of B19V DNA were available from 53 Dutch patients, mainly children with symptoms of fifth disease. The B19V sequences of these patients were all genotype 1a and also showed a division into the two groups at synonymous positions (Figure 4). When the B19V sequences of the patients were compared to the B19V sequences of the blood donors, no distinction between patient and donor strains was found. Thus, the B19V groups 1a1 and 1a2 are present in Dutch patients with fifth disease and in asymptomatically infected blood donors.

### Spatio-temporal Distribution of B19V Subtypes in the Netherlands

When the sequenced B19V groups 1a1 and 1a2 of Dutch blood donors were plotted on a geographical map, for each year the strains were randomly distributed over the Netherlands (Figure S1). The B19V groups 1a1 and 1a2 don’t appear to have a specific spatial distribution in the Netherlands in the years 2003–2009. The relative number of B19V 1a1 and 1a2 infections in Dutch blood donors was different in 2003 and 2004, with more 1a1 (79% and 64% respectively) found than 1a2 (21% and 36%). In later years (2006–2009) the group distribution was 50–50 percent among blood donors (Figure 5A). In the Dutch patients the 1a1/1a2 distribution was approximately 50–50 percent in 2003–2006, but the 1a2 frequency increased over time, to 85% in 2008–2009 (Figure 5B). However, the numbers of sequences are very small in these years. In conclusion B19V group 1a2 currently may be increasing its prevalence at the expense of group 1a1.

## Discussion

For this report we studied the molecular epidemiology of B19V, comparing B19V sequences from Dutch asymptomatic blood donors, from Dutch patients with fifth disease, and from GenBank. In both donors and patients we only identified B19V genotype 1a, which indeed is the most prevalent genotype in northern Europe [1]. Phylogenetic analysis showed that genotype 1a comprises two groups, named 1a1 and 1a2. The occurrence of 1a1 and 1a2 in healthy blood donors and in patients suggests that the two groups do not differ in virulence. Comparison of the 1a1 and 1a2 consensus sequences to B19V sequences from GenBank showed that B19V subtypes 1a1 and 1a2 occur around the world.

The 1a1 and 1a2 groups were randomly dispersed across the Netherlands, in different years. However, over time the relative number of 1a2 may be increasing. In hindsight, a gradual replacement of 1a1 with 1a2 also occurred in Japan. During six consecutive outbreaks of fifth disease in Sapporo, a B19V subgroup, most similar to AY504945 (GB_2003) or 1a1 in our study, was dominant from 1993–1997; and was replaced from 1998 onwards by a subgroup, which is most similar to AJ781038 (DE_2004) or 1a2 in our study [12]. Probably 1a2 arose later than 1a1, as suggested by a comparison of our data to previous studies, which analysed B19V using restriction enzyme analysis. When 75% consensus sequences of 1a1 and 1a2 are digested ‘in silico’, using the restriction enzymes as published by Mori et al [21] and Umene et al [22], the 1a1 group restriction profile is similar to the historical ‘group-II’ restriction profile [16]. However, the restriction pattern of the present 1a2 group was not described previously, it contains an extra HindIII site and lacks the BamHI site, as compared to 1a1 [21], [23], [24]. Japan may be the origin of the 1a2 subgroup since the earliest 1a2 sequences are from this country; however this may be biased due to the small number of 1a2 sequences available in GenBank. To follow whether 1a2 will replace 1a1, B19V strains from the coming years will have to be analysed. Since the B19V 1a1 and 1a2 groups are found worldwide we propose that these two groups are called subtypes of B19V genotype 1a.

To investigate whether the nucleotide differences between the two subgroups resulted in amino acid differences, we have analysed the synonymous and nonsynonymous mutations. The dS/dN ratios found for the 2 subgroups were >1 which indicates that there is negative selection in the 1a1 and 1a2 groups. This is in agreement with previous data for *Parvoviridae* [25]. However, the dS/dN ratio should be interpreted with care since this analysis was performed in one species and the dS/dN was originally developed to compare sequences from divergent species [26]. In addition, there don’t appear to be functional differences in the subgroups in ability to cause disease. A remarkable finding of this study is that two groups of parvovirus co-exist around the world, each showing a distinct pattern of genome-wide synonymous mutations, with only few nonsynonymous mutations. Many virus species, including the major human pathogens human immunodeficiency virus (HIV), hepatitis C virus (HCV), and hepatitis B virus (HBV), consist of genetically different lineages, called serotypes, genotypes, subtypes, or clades. These lineages are defined by nonsynonymous mutations, with practical consequences for diagnostic procedures, antiviral therapy and vaccine development [27], [28]. In addition, the amino acid differences often reflect biological differences between different strains of these viruses [28]. Although synonymous differences are not 100% neutral and may be under evolutionary pressures related to nucleotide preferences and codon usage [29]–[32], we found no evidence for these two evolutionary factors being involved in the differences between the two virus lineages. Still synonymous differences can affect mRNA stability, mRNA structure, splicing, and even protein folding, thus we cannot fully exclude biological differences between parvovirus subgroups 1a1 and 1a2. However, the synonymous mutations in B19V subtypes 1a1 and 1a2 are distributed throughout the whole parvovirus genome. Possibly, in the absence of nucleotide preferences and codon bias, the two subtypes are the result of an evolutionary bottleneck.

## Supporting Information

Figure S1 **Map showing the geographical distribution of B19V 1a1 and 1a2 sequences found in Dutch blood donors in the years 2004, 2006 and 2009.**
(TIF)
