# Supplementary figures and images for: Global Co-Existence of Two Evolutionary Lineages of Parvovirus B19 1a, Different in Genome-Wide Synonymous Positions
Source: PLoS One. 2012 Aug 13;7(8):e43206. doi: 10.1371/journal.pone.0043206 (PMC3418230; doi:10.1371/journal.pone.0043206)

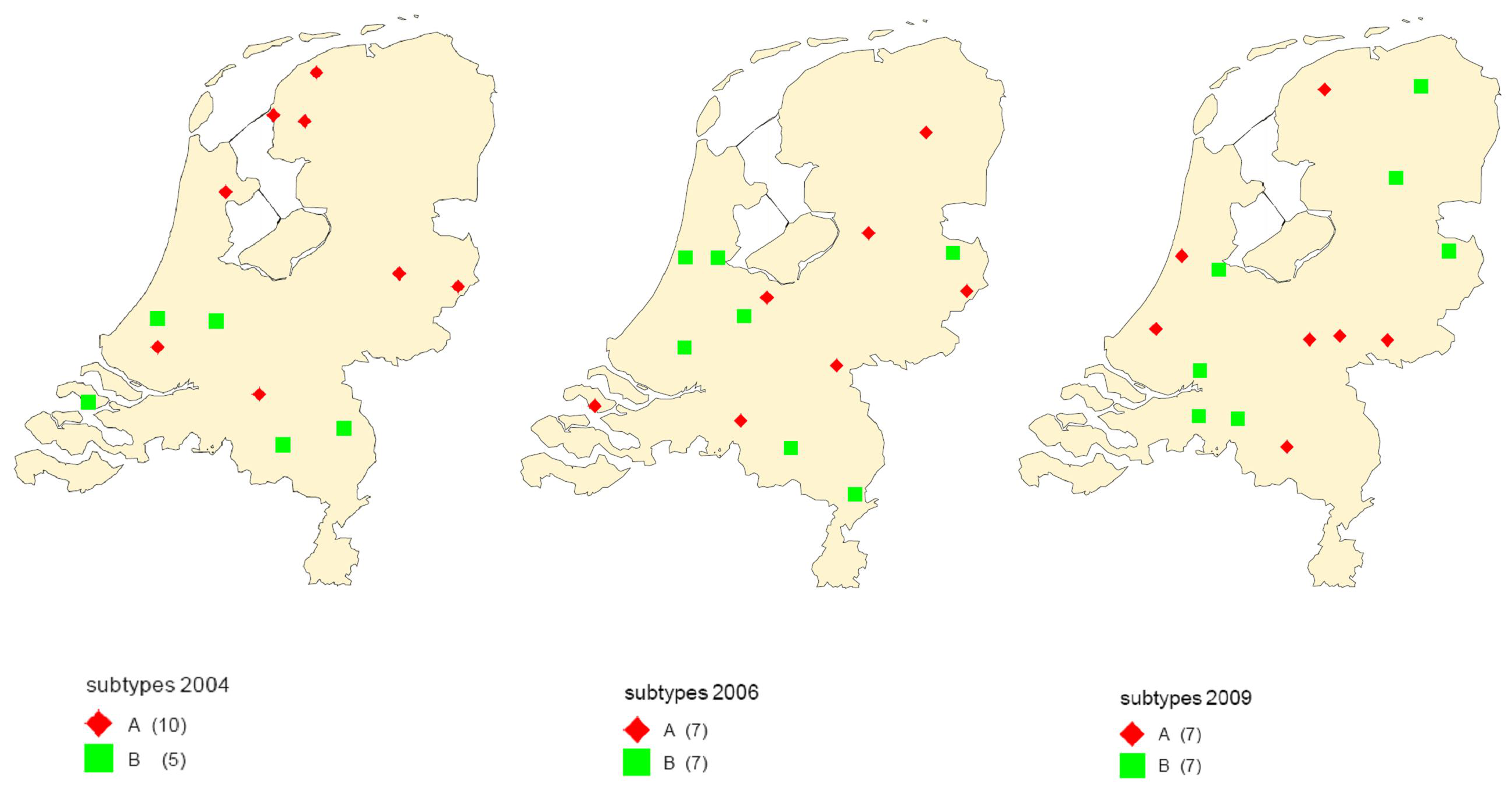

Supplement: Figure S1 — Map showing the geographical distribution of B19V 1a1 and 1a2 sequences found in Dutch blood donors in the years 2004, 2006 and 2009. (TIF) [file pone.0043206.s001.tif]
